# HDAC8 suppresses the epithelial phenotype and promotes EMT in chemotherapy-treated basal-like breast cancer

**DOI:** 10.1186/s13148-022-01228-4

**Published:** 2022-01-11

**Authors:** Garyfallia Pantelaiou-Prokaki, Iga Mieczkowska, Geske E. Schmidt, Sonja Fritzsche, Evangelos Prokakis, Julia Gallwas, Florian Wegwitz

**Affiliations:** 1 Department of Gynecology and Obstetrics, University Medical Center Göttingen, Göttingen, Germany; 2 Translational Molecular Imaging, Max Planck Institute for Experimental Medicine, Göttingen, Germany; 3 Department of General, Visceral and Pediatric Surgery, University Medical Center Göttingen, Göttingen, Germany; 4 Department of Gastroenterology, GI-Oncology and Endocrinology, University Medical Center Göttingen, Göttingen, Germany

**Keywords:** HDAC8, MET, EMT, BLBC, TNBC, Chemotherapy, Epigenetics, H3K27ac, Epithelial transcription factors

## Abstract

**Background:**

Basal-like breast cancer (BLBC) is one of the most aggressive malignant diseases in women with an increased metastatic behavior and poor prognosis compared to other molecular subtypes of breast cancer. Resistance to chemotherapy is the main cause of treatment failure in BLBC. Therefore, novel therapeutic strategies counteracting the gain of aggressiveness underlying therapy resistance are urgently needed. The epithelial-to-mesenchymal transition (EMT) has been established as one central process stimulating cancer cell migratory capacity but also acquisition of chemotherapy-resistant properties. In this study, we aimed to uncover epigenetic factors involved in the EMT-transcriptional program occurring in BLBC cells surviving conventional chemotherapy.

**Results:**

Using whole transcriptome data from a murine mammary carcinoma cell line (pG-2), we identified upregulation of *Hdac4*, *7* and *8* in tumor cells surviving conventional chemotherapy. Subsequent analyses of human BLBC patient datasets and cell lines established HDAC8 as the most promising factor sustaining tumor cell viability. ChIP-sequencing data analysis identified a pronounced loss of H3K27ac at regulatory regions of master transcription factors (TFs) of epithelial phenotype like *Gata3*, *Elf5*, *Rora* and *Grhl2* upon chemotherapy. Interestingly, impairment of HDAC8 activity reverted epithelial-TFs levels. Furthermore, loss of HDAC8 activity sensitized tumor cells to chemotherapeutic treatments, even at low doses.

**Conclusion:**

The current study reveals a previously unknown transcriptional repressive function of HDAC8 exerted on a panel of transcription factors involved in the maintenance of epithelial cell phenotype, thereby supporting BLBC cell survival to conventional chemotherapy. Our data establish HDAC8 as an attractive therapeutically targetable epigenetic factor to increase the efficiency of chemotherapeutics.

**Graphical abstract:**

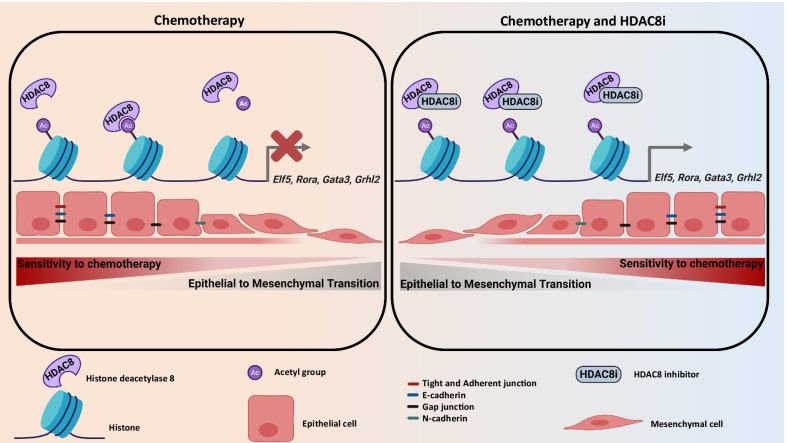

**Supplementary Information:**

The online version contains supplementary material available at 10.1186/s13148-022-01228-4.

## Background

Triple-negative breast cancer (TNBC) is considered one of the most aggressive breast cancer (BC) subtypes. TNBC accounts for 10–15% of all BC and tends to be more common in younger women (under 40 years old) with an African-American origin bearing a *BRCA1* mutation [1]. TNBCs distinguish themselves from other histological BC subtypes by the absence of estrogen receptor (ER), progesterone receptor (PR) expression and human epidermal growth factor 2 (HER2) gene amplification, as assessed by immunohistochemistry (IHC) and fluorescence in situ hybridization (FISH), respectively [2]. This characteristic makes the group of TNBC lesions largely overlapping with the basal-like (BLBC) and to a much lower extend with normal-like molecular subtypes [1, 3], thereby rendering this type of malignancies insensitive to hormone and anti-HER2-targeted therapies. Consequently, therapeutic options for TNBC are relatively scarce, mostly limited to surgical removal of the lesion eventually combined with radiotherapy and/or chemotherapy. Here, anthracycline, cyclophosphamide, taxanes or platinum salts are most commonly selected and often used in combination [3, 4]. Unfortunately, the efficiency of such therapies frequently declines because of adaptations of the tumor cells and rapid acquisition of chemoresistant traits. Patients with relapses or residual disease have a high probability to develop metastatic outgrowth and subsequently succumb to their disease [2].

Epithelial–mesenchymal transition (EMT) and the reverse mesenchymal-to-epithelial transition (MET) are both indispensable for various processes in multicellular organisms, like during embryogenesis, wound repair, placentation or inflammation [5–7]. However, increasing evidence established a strong implication of the EMT also in the phenomenon of therapy resistance, cancer cell dissemination and distant organ colonization [8–10]. Interestingly, deep involvement of epigenetic mechanisms in the cellular plasticity of tumor cells is nowadays well established [11]. Due to the reversible nature of these pathological alterations, targeting epigenetic factors controlling EMT and MET processes represent an interesting strategy to revert aggressive chemotherapy-resistant metastatic phenotypes [12]. Histone deacetylases (HDACs) are epigenetic effectors categorized in four classes based on their structure, enzymatic function and subcellular localization. HDACs enact a major gene silencing role to regulate multiple gene expression programs involved in, among others, inflammation, cell proliferation, cancer stemness and EMT processes via de-acetylating histones [13].

The WAP-T mammary carcinoma mouse model was designed to investigate processes involved in the progression and metastasis of basal-like malignancies (22–27). We previously reported that WAP-T mammary carcinomas cells treated with conventional cytotoxic therapy develop strategies to survive the treatment in vivo. Interestingly, we identified in the surviving tumor cells pronounced mesenchymal and stem cells specific features that are characteristic for more aggressive phenotypes. These cells showed an increased tendency to disseminate to distant organs (28). To get a better understanding of the mechanisms allowing dynamic gene expression changes responsible for the EMT-driven refractory response to chemotherapy, we designed a combined high-throughput mRNA- and ChIP-sequencing (mRNA-seq and ChIP-seq) approach and analyzed epigenetic changes occurring in a murine mammary carcinoma cell line (pG-2) surviving conventional cytotoxic chemotherapy. We identified three upregulated HDACs, *Hdac4*, *Hdac7* and *Hdac8.* Interestingly, BLBC patients with high HDAC8 expression demonstrated the poorest prognosis. Our results showed that HDAC8 promotes EMT program and therapy resistance by inhibiting the expression of gatekeeper transcription factors of the epithelial phenotype (*Elf5*, *Gata3*, *Rora* and *Grhl2*). Accordingly, the combination of conventional chemotherapies with HDAC8 silencing or HDAC8-inhibition considerably potentiated the therapeutic effectiveness. In summary, we identified HDAC8 as a promising targetable epigenetic regulator to enhance the effectiveness of BLBC treatments.

## Results

### Chemotherapy induces EMT in murine and human BLBCs

**Fig. 1:**
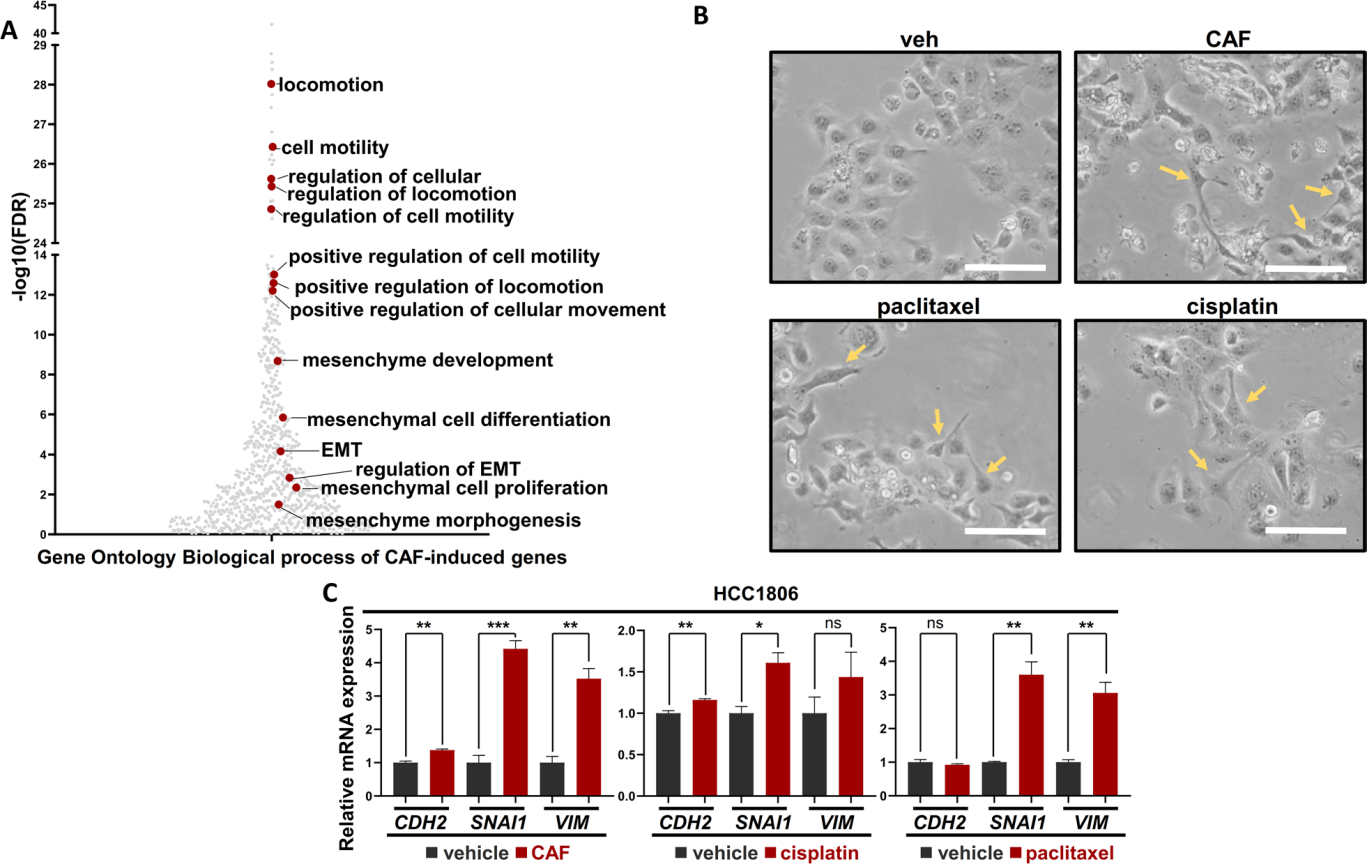
Chemotherapy induces EMT in murine and human BLBC cells. **A** Scatter plot showing EMT signatures, using the online pathway enrichment analysis tool gProfiler, on pG-2 cells upon CAF treatment for 48 h.** B** Brightfield pictures of HCC1806 cells showing a mesenchymal phenotype upon different chemotherapies (CAF, cisplatin and paclitaxel) for 48 h. The yellow arrows indicate cells with pronounced mesenchymal morphology. White scale bars = 50 µm, **C** Quantitative real-time PCR (qRT-PCR) on EMT-related genes in vehicle- and CAF-, cisplatin- or paclitaxel-treated (for 48 h) HCC1806 cells. All experiments were performed in biological triplicate. ns = not significant, *p-val < 0.05, **p-val < 0.01, ***p-val < 0.005. **C** Student's *t* test. Error bars are standard error of the mean (SEM)

In the past, we and others have shown that BLBC cells activate the EMT transcriptional program to survive conventional chemotherapy [14–16]. In a recent study, we leveraged the WAP-T mammary carcinoma mouse model system (parental G-2 cells; pG-2) to study the mechanisms underlying resistance to combined cytotoxic chemotherapy (cyclophosphamide, adriamycin, 5-fluorouracil; short CAF) and confirmed the enrichment of EMT-associated features along with the disease progression (Fig. 1A), [17, 18]. Hence, we sought to validate the activation of the EMT transcriptional program in a human BLBC cell line (HCC1806) upon different conventional chemotherapy treatments (CAF, cisplatin and paclitaxel). Indeed, we observed changes of cell morphology typically observed during the acquisition of mesenchymal traits in treated cells (Fig. 1B). Furthermore, an increase of typical EMT markers *CDH2*, *SNAI1* and *VIM* expression was observed in all treated cells, confirming the activation of the EMT-transcriptional program upon chemotherapy (Fig. 1C).

### BLBC cells surviving chemotherapy upregulates HDAC4, HDAC7 and HDAC8

**Fig. 2:**
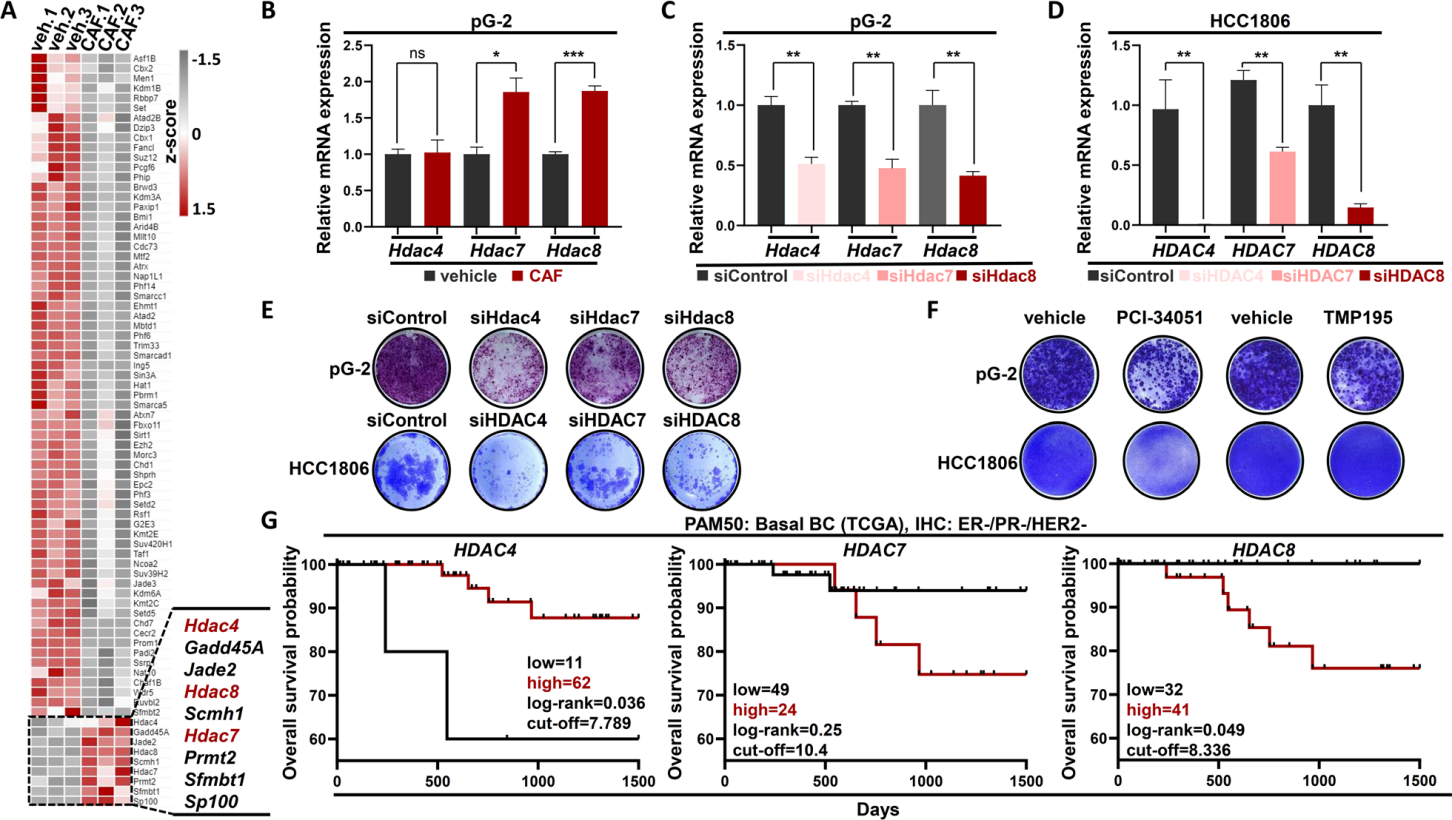
HDAC4, HDAC7 and HDAC8 are upregulated in CAF-treated pG-2 and in CAF-resistant clones. **A** Heatmap of differentially regulated epigenetic factors in pG-2 cells upon CAF treatment (48 h) (basemean ≥ 15, p-adj < 0.05, log2FC ≥|0.7|). **B** qRT-PCR of *Hdac4, Hdac7, Hdac8* in vehicle- and CAF-treated (48 h) pG-2 cells. **C**, **D** qRT-PCR validating the efficiency of *HDAC4*, *HDAC7* and *HDAC8* knockdowns in pG-2 (**C**) and HCC1806 (**D**), respectively. **E** Proliferation assay of pG-2 and HCC1806 cells upon HDAC4*,* HDAC7 *and* HDAC8 silencing, assessed by crystal violet staining. **F** Proliferation assay of pG-2 and HCC1806 cells upon HDAC8 inhibition (5 μM and 20 μM PCI34051, respectively) or HDAC4/HDAC7 inhibition (4 μM and 2 μM TMP195, respectively), assessed by crystal violet staining. **G** Kaplan–Meier plots showing the overall survival probability of *HDAC4-*, *HDAC7-* and *HDAC8-*expressing BLBC patients (expression and survival data are from the TCGA-BRCA database). All experiments were performed in triplicate. ns = not significant, *p-val < 0.05, ***p-val < 0.005. Statistical test: **B** Student's *t* test, error bars: standard error of the mean (SEM); **G** Log-rank test

Previous studies of our group and others identified that epigenetic mechanisms are frequently involved in the EMT transcriptomic program induction of cancer cells [18–22]. To better understand factors that could be responsible for the transcriptional changes observed in BLBC cells upon chemotherapy treatment and to identify potentially druggable upregulated epigenetic effectors, we re-analyzed the mRNA-seq data of our previous study [18]. We identified 76 differentially regulated epigenetic factors (log2FC ≥ l0.7l, *p*-adj < 0.05), of which only 9 were significantly upregulated (Fig. 2A). A closer look at these factors identified a group of three histone deacetylases (*Hdac4, Hdac7, Hdac8*) with reported implications in cancer patient's prognosis and with a wide range of available specific inhibitory compounds [23, 24]. qRT-PCR confirmed the increased expression of *Hdac7, Hdac8* upon CAF treatment in pG-2 cells (Fig. 2B). To study the role of *Hdac4, Hdac7* and *Hdac8* on the viability of murine pG-2 and human HCC1806 cells, we performed siRNA-mediated knockdown for every single gene (Fig. 2C, D). Interestingly, all siRNA treatments were detrimental for pG-2 cells, siHdac4 and siHdac8 showing the strongest phenotype impairment (Fig. 2E and Additional file 1: Fig. S1A). We observed a similar strong impairment of HCC1806 cell proliferation upon HDAC4 and HDAC8 loss (Fig. 2E and Additional file 1: Fig. S1B). To next test whether the enzymatic activities of these HDACs play a role in tumor cell fitness, we treated pG-2 and HCC1806 cells with specific inhibitors for HDAC8 (PCI-34051) and HDAC4/7 (TMP195) and subsequently assessed tumor cell proliferation. Interestingly, loss of HDAC4 and 7 activity impaired pG-2 cells (Fig. 2F, Additional file 1: Fig. S1C and S1F) and was not able to inhibit HCC1806 growth (Fig. 2F and Additional file 1: Fig. S1D). In contrast, PCI-34051 treatment robustly impaired the proliferation of both cell lines (Fig. 2F, Additional file 1: Fig. S1C-E). To test whether the combination of both HDAC8 and HDAC4/7 could increase the sensitivity of the cells to the treatment, we simultaneously treated pG-2 cells with increasing doses of both inhibitors. Here, we could not identify any improved sensitization with the combination therapy. On the contrary, the anti-proliferative effects of the PCI-34051 inhibitor (5–10 μΜ) in pG-2 cells were attenuated with increasing doses of TMP195 (Additional file 1: Fig. S1G). To next estimate if the identified *HDAC4*, *HDAC7* and *HDAC8* play a potential role in BLBC patient outcome, we analyzed data from the Breast Cancer Adenocarcinoma (BRCA) dataset from The Cancer Genome Atlas (https://portal.gdc.cancer.gov/) and the Kaplan–Meier Plotter database (www.kmplot.com). In line with our results in vitro, HDAC8 was the only member of the three selected HDACs whose expression significantly correlated with a poor BLBC patient prognosis (Fig. 2G and Additional file 1: Fig. S1H). Taken together, our results suggest an important role of HDAC8 in murine and human BLBC aggressiveness.

### HDAC8 supports the induction of EMT by suppressing the MET pathway in BLBC cells surviving chemotherapy

HDAC8 is a well-studied deacetylase exerting a function on numerous histone and non-histone substrates involved in various cellular homeostatic mechanisms. Structural Maintenance of Chromosomes 3 (SMC3), a subunit of the cohesin complex, represents one of the best characterized target for deacetylation by HDAC8 [24]. Therefore, we reasoned that HDAC8 could enact its pro-survival function by deacetylating SMC3, thereby facilitating the progression of the cell cycle as proposed by Dasgupta et al. [25]. Surprisingly, chemotherapy treatment of pG-2 cells did not led to a reduction of total acetylated SMC3 (ac-SMC3), as assessed by western blot (Additional file 1: Fig. S1I). Furthermore, PCI-34051 treatment at concentrations showing tumor cell phenotype impairment (5–10 μΜ) did not result in a further increase of ac-SMC3 neither in basal growth conditions nor upon chemotherapy (Additional file 1: Fig. S1I). Therefore, we concluded that HDAC8 function in BLBC cell fitness is rather independent of SMC3.

**Fig. 3:**
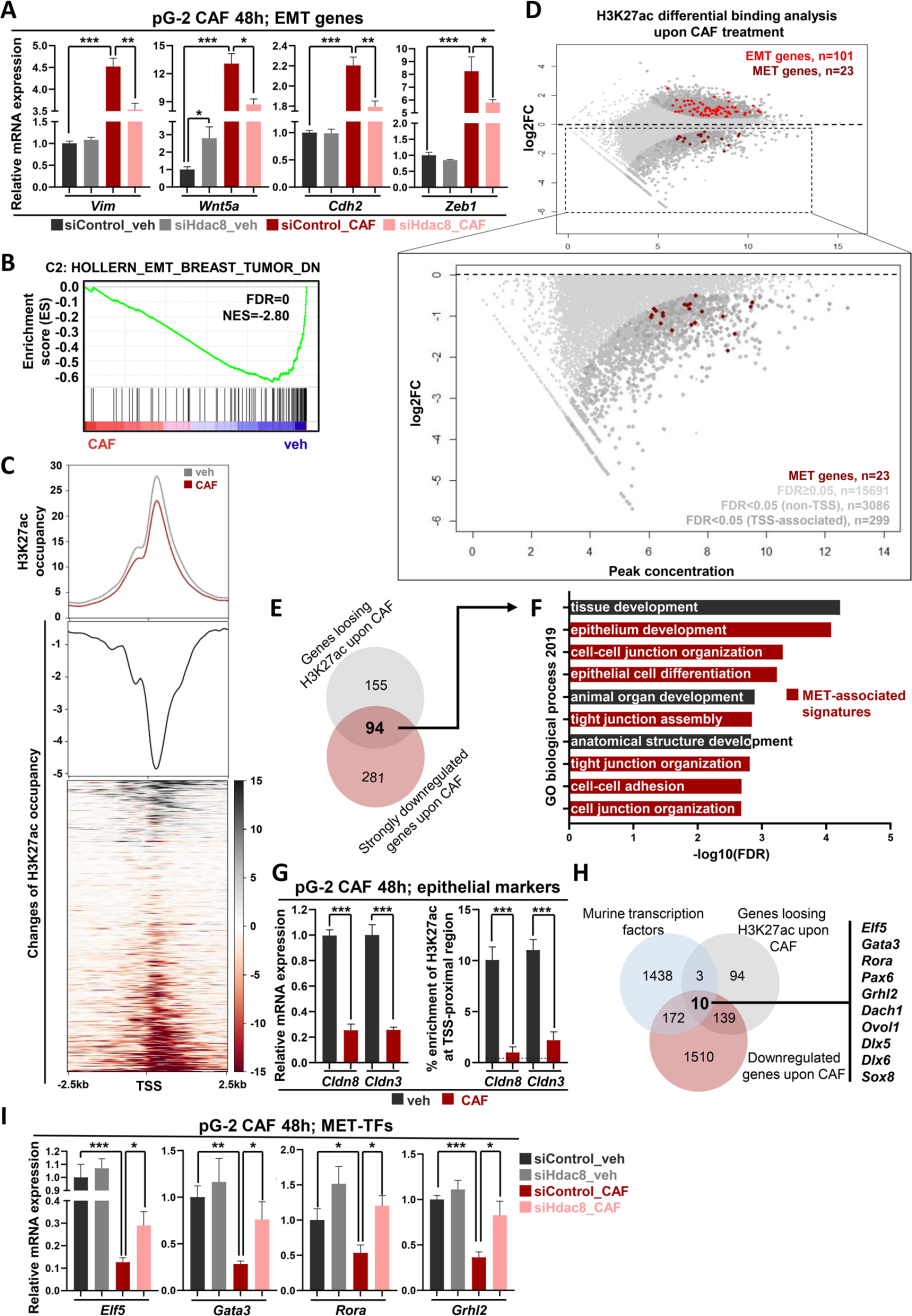
HDAC8 supports the induction of EMT by suppressing key-MET TFs in BLBC cells surviving cytotoxic therapy. **A** Assessment of *Vim*, *Wnt5a*, *Cdh2, Zeb1* mRNA expression levels by qRT-PCR in vehicle- or CAF-treated (48 h) pG-2 cells, with or without *Hdac8* silencing. **B** GSEA profile showing an enrichment of the "HOLLERN_EMT_BREAST_TUMOR_DN" signature (MSigDB: C2 curated gene sets) in pG-2 cells at basal state (veh) compared to CAF-treated. NES: Normalized Enrichment Score. **C** Aggregate profile showing changes of H3K27ac at TSS regions of strongly downregulated genes in vehicle- and CAF-treated pG-2 cells (normalized counts basemean ≥ 15, *p*-adj < 0.05, log2FC ≤ -− 1.5). **D** Differential binding analysis (DiffBind) depicting changes of H3K27ac occupancy between vehicle- and 48 h CAF-treated pG-2 cells. Genomic regions showing significant changes of H3K27ac occupancy at EMT- or MET-associated genes (MSigDB C2, see method section) were labeled with light red and dark red dots, respectively. **E** Venn diagram showing genes simultaneously downregulated (normalized counts basemean > 15, p-adj < 0.05, log2FC < − 1.5) and loosing H3K27ac at TSS regions (Diffbind parameters: conc > 3, *p*-adj < 0.05, FC < -0.5) upon CAF treatment in pG-2 cells. **F** Pathway enrichment analysis showing that genes with loss of expression and H3K27ac enrich for MET- and cell differentiation-associated signatures (labeled in red). Analysis performed with the online tool. **G** qRT-PCR and ChIP-RT-PCR of epithelial-specific markers (*Cldn8*, *Cldn3*) in vehicle- and CAF-treated pG-2 cells*.* **H** Venn diagram showing 10 transcription factors (TFs) with simultaneous gene expression (normalized counts basemean > 15, *p*-adj < 0.05, log2FC < -− 0.7) and H3K27ac occupancy loss (Diffbind parameters: conc > 3, *p*-adj < 0.05, FC < − 0.5) upon CAF treatment in pG-2 cells. The murine transcription factor list was retrieved from the Animal *TFDB3.0* TF database. **I** qRT-PCR assessing changes of MET-TFs *Elf5*, *Gata3*, *Rora*, *Grhl2* upon pG-2 CAF treatment for 48 h, w/ or w/o *Hdac8* silencing. Statistical test: **A**, **I** one-way ANOVA, **G** Student's *t* test. *p-val < 0.05, **p-val < 0.01, ***p-val < 0.005. Error bars are standard error of the mean (SEM)

Since our previous results showed that HDAC8 possesses tumor-supportive properties in BLBC cells, we posited that these effects may result from its ability to positively influence the EMT transcriptional program in chemotherapy surviving cells. To test this hypothesis, we assessed the expression of well-known EMT-drivers (*Vim*, *Cdh2*, *Wnt5a, Zeb1*) in pG-2 cells upon CAF treatment and/or *Hdac8* knockdown via qRT-PCR (Fig. 3A and Additional file 1: Fig. S2A). As expected, chemotherapy treatment induced their expression. However, loss of HDAC8 strongly reverted the induction of these genes upon chemotherapy treatment. Under basal growth conditions, only the expression of *Wnt5a* was affected by HDAC8 knockdown. The EMT process relies on one hand on the activation of EMT-TFs and effectors, but also on the repression of epithelial genes capable of inhibiting or reversing this process. Through its enzymatic activity, HDAC8 removes acetyl groups among others from histones, leading to gene inactivation in the deacetylated regions [26]. Therefore, we hypothesized that HDAC8 may support the induction of the EMT-transcriptional program by repressing genes driving epithelial phenotype (MET genes). To test this assumption, we examined whether genes commonly downregulated during EMT were also downregulated in chemotherapy-treated cells. Indeed, Gene Set Enrichment Analyses (GSEA) supported this assumption (Fig. 3B). Furthermore, by analyzing our ChIP-seq data from pG-2 cells surviving 48-h CAF treatment, we observed that the majority of strongly downregulated genes (log2FC ≤ − 1.5) upon chemotherapy showed a pronounced loss of H3K27ac in their TSS-proximal region (Fig. 3C). To assess whether HDAC8 is involved in the repression of these genes, we determined all H3K27ac occupied regions and investigated the changes of H3K27ac occupancy in pG-2 cells upon CAF treatment via Differential Binding (DiffBind) analysis. Pathway enrichment analysis of TSS-proximal regions losing H3K27ac using the Genomic Regions Enrichment of Annotations Tool (GREAT) showed a strong enrichment for tight junction and cell-to-cell adhesion signatures. This result supports that genes characteristic for epithelial cell phenotype maintenance markedly lose H3K27ac in their regulatory region upon CAF treatment (Additional file 1: Fig. S2B). A closer analysis of the DiffBind results identified a great number of EMT genes with increased H3K27ac (*n* = 101 EMT genes) and several MET genes (*n* = 23 MET genes) with a loss of H3K27ac at their TSS-proximal region (Fig. 3D). We decided to focus on the same strongly downregulated gene cohort (Fig. 3C) with concomitant loss of TSS-proximal H3K27ac enrichment upon CAF treatment (*n* = 94 genes, Fig. 3E) to perform a pathway enrichment analysis on these genes using the online tool ‘’Enrichr’’ (https://maayanlab.cloud/Enrichr/). Strikingly, this particular group of genes was strongly enriched with epithelial differentiation signatures (Fig. 3E, F). Tight junctions were the most prominently lost class of factors in the different MET signatures upon chemotherapy treatment. To validate the regulation of the identified gene signature, we selected two tight junction genes *(Cldn3*, *Cldn8*) and confirmed the loss of TSS-proximal H3K27ac and their decreased gene expression in chemotherapy-treated pG-2 cells by ChIP-qPCR and qRT-PCR, respectively (Fig. 3G, Additional file 1: Fig. S2C).

Similar to EMT, the MET-driven cell differentiation is a well-documented transcriptional program involving MET-committed transcription factors (MET-TFs) in various biological contexts [27–29]. Hence, we sought to identify MET-TFs potentially under control of HDAC8 repression, therefore showing decreased promoter-proximal H3K27ac occupancy with a concomitant gene expression loss upon CAF treatment. We identified 10 downregulated TFs showing a prominent decrease of TSS-proximal H3K27ac occupancy upon CAF treatment, some of those with a well-established functional role in epithelial cell differentiation [29–36] (Fig. 3H, Additional file 1: Fig. S2D–E). To next assess the potential repressive function of HDAC8 on these genes, we performed a knockdown and a PCI-34051-mediated inhibition of HDAC8 in pG-2 cells with or without CAF treatment. We selected four MET-TF genes and confirmed their decreased expression upon chemotherapy alone, as expected from our mRNA-seq results (Fig. 3I). Strikingly, both HDAC8 knockdown or inhibition partially rescued the expression levels of the four MET-TFs (*Elf5*, *Gata3*, *Rora* and *Grhl2)* tested here (Fig. 3I and Additional file 1: Fig. S2H), but also rescued the expression of epithelial-specific markers (*Cdh1*, *Cldn3*, *Cldn8*) (Additional file 1: Fig. S2F and S2G). Of note, inhibition of HDAC4 and HDAC7 by TMP195 alone or in combination with PCI-34051 could not restore the expression of MET-TFs and epithelial markers in chemotherapy-treated in p-G2 cells to the same extent as PCI-34051 alone. Concordantly, mesenchymal markers vimentin (*Vim*) and *Zeb1* were efficiently suppressed only in PCI34051-treated cells upon chemotherapy and not by single or co-treatment with TMP195 (Additional file 1: Fig. S2I). Finally, to further exclude SMC3’s potential involvement in the regulation of this MET transcriptional signature, we re-analyzed a publically available microarray gene expression dataset (GSE38252) and confirmed that MCF7 BC-cells do not regulate the expression of any of these genes upon SMC3 knockdown (Additional file 1: Fig. S2J). Therefore, our findings strongly support a transcriptional repressive function of HDAC8 exerted on the MET program.

### High HDAC8 expression correlates with repression of epithelial differentiation program in BLBC patients

**Fig. 4:**
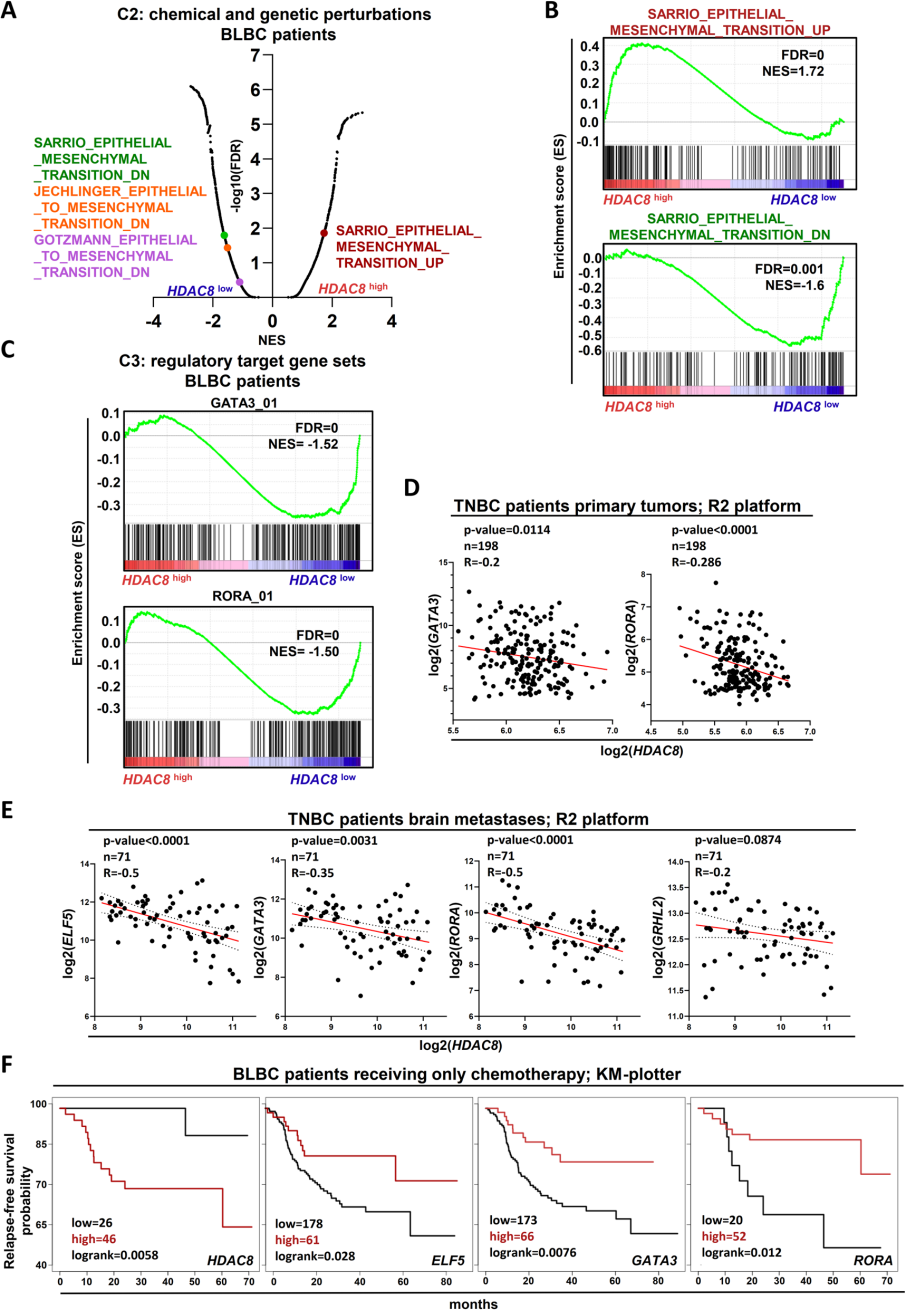
High *HDAC8* expression correlates with repression of the epithelial differentiation program in BLBC patients. **A** Plot depicting all C2 curated gene sets (MSigDB) enriched in GSEA analyses of *HDAC8*^high^- and *HDAC8*^low^-expressing BLBC patients. **B** Representative gene set enrichment profiles of the EMT and MET transcription programs in HDAC8^low^- and HDAC8^high^-BLBC patients. NES: Normalized Enrichment Score. **C** Gene set enrichment profiles of the MET-TFs GATA3 and RORA whose gene targets are particularly expressed in *HDAC8*^*l*ow^-BLBC patients. **D–E** Scatter plot showing the anti-correlation of the MET-TFs with *HDAC8* in primary tumors (**D**) and brain metastases (**E**) of TNBC patients. Data were retrieved from the R2 platform. **F** Kaplan–Meier plots showing the relapse-free survival probability of *HDAC8-, ELF5-, GATA3-* and *RORA-*expressing BLBC patients receiving only chemotherapy as a treatment option (Log-rank test). Data were retrieved from the KM plotter. **A–C** Source of patient gene expression data: TCGA. Source of gene set enrichment profiles: MSigDB

To corroborate our results with the clinical situation of BLBC patients, we analyzed the gene expression profiles of low and high *HDAC8*-expressing lesions from the BRCA-TCGA dataset. As suggested by our previous in vitro investigations, we observed a significant enrichment of EMT gene expression signatures in *HDAC8*^high^ patients, whereas *HDAC8*^low^ lesions-enriched gene sets generally downregulated upon activation of the EMT program (Fig. 4A, B). Further analyses of this patient collective on the C3 library (MSigDB) identified that *HDAC8*^low^ patients enrich for genes with at least one binding element for *GATA3* or *RORA* in their regulatory region (Fig. 4C). These last results point at a possible higher activity of MET-TFs in *HDAC8*^low^ BLBC-lesions. We reasoned that if HDAC8 represses MET-TFs also in BLBC-patients, a negative correlation between HDAC8- and MET-TFs should be detectable. Therefore, we retrieved expression data from the R2-platform. Here, *HDAC8* expression negatively correlated with the expression of *GATA3* and *RORA* in primary TNBC tumors (Fig. 4D). Accordingly, further analyses on brain metastases of TNBC patients confirmed a significant negative correlation of *HDAC8* with *ELF5*, *GATA3*, *RORA* and a mild but insignificant negative correlation with *GHRL2* (Fig. 4E). Finally, we asked whether these particular MET-TFs have a prognostic value for BLBC patients' outcomes. Interestingly, whereas *HDAC8*^high^-patients had the highest probability of relapse, *ELF5*^high^-, *GATA3*^high^- and *RORA*^high^-patients displayed the lowest relapse rate (Fig. 4F). Together, these data strongly indicate a tumor-promoting role of HDAC8 through a repressive function on the MET program in BLBC patients.

### HDAC8 silencing or inhibition sensitizes BLBC cells to chemotherapy treatment

**Fig. 5:**
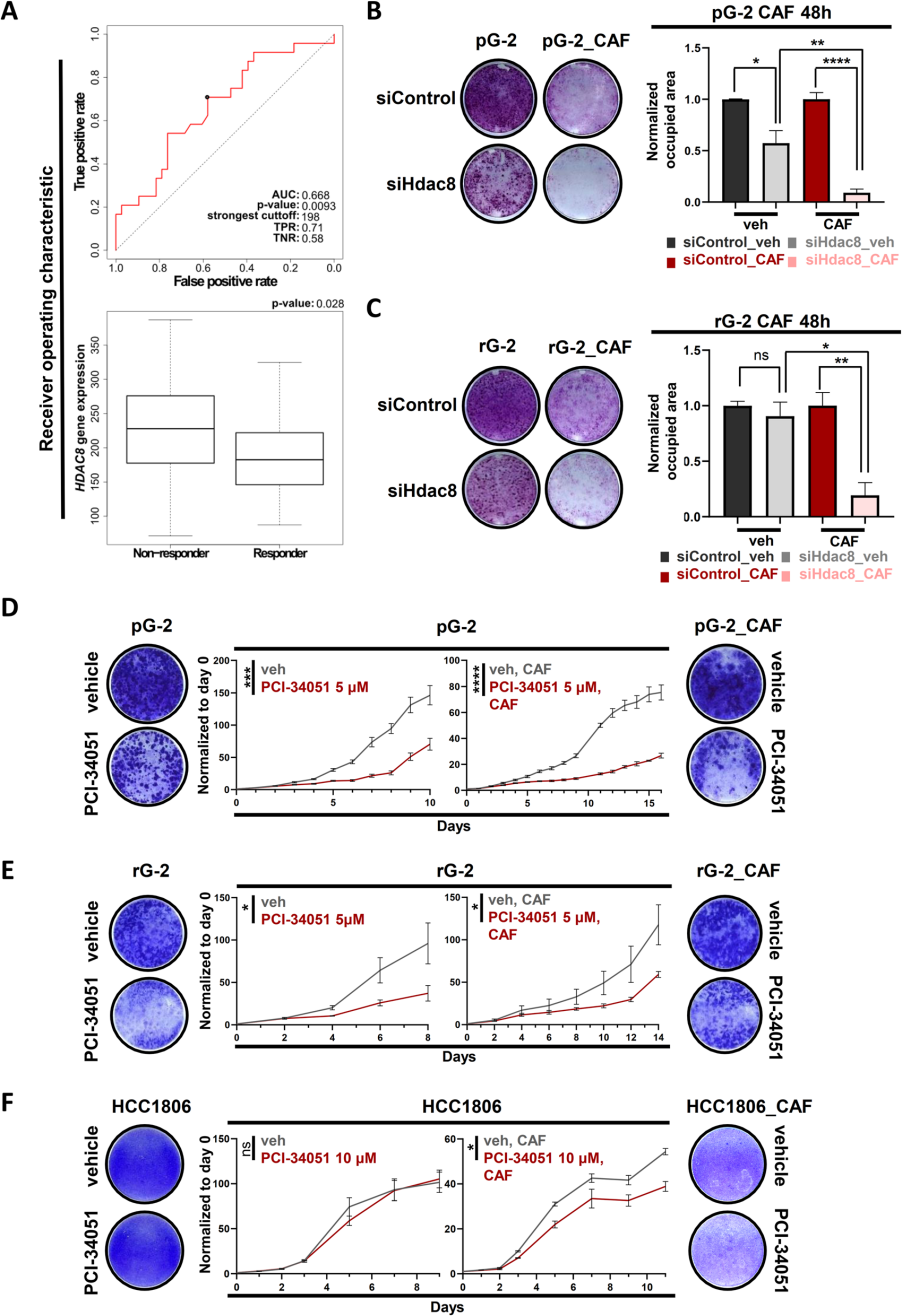
HDAC8 silencing or inhibition sensitizes BLBC cells to chemotherapy treatment. **A** ROC analysis from publically available TNBC data demonstrating that patients with poor response to chemotherapy harbor high expression levels of *HDAC8.* Box plots: Mann–Whitney test. **B, C** Proliferation assay of CAF-treated pG-2 (**B**) and rG-2 (**C**) cells upon *Hdac8* silencing. Representative crystal violet pictures are at the right panel of the bar charts. **D** Proliferation assay of pG-2 treated with low CAF doses (156.25 ng/ml cyclophosphamide, 7.8 ng/ml doxorubicin, 156.25 ng/ml 5-FU) for 48 h, w/ or w/o HDAC8 inhibition (5 μM PCI34051). **E** rG-2 cells treated with CAF for 48 h, w/ or w/o HDAC8 (5 μM PCI34051). Representative crystal violet pictures are at the left and right panel of proliferation kinetic graphs for pG-2 and rG-2, respectively. **F** Proliferation assay of HCC1806 treated with low CAF doses (156.25 ng/ml cyclophosphamide, 7.8 ng/ml doxorubicin, 156.25 ng/ml 5-FU) for 48 h, w/ or w/o HDAC8 inhibition (10 μM PCI34051). All experiments were performed in biological triplicates. ns = no difference, *p-val < 0.05, ***p-val < 0.005, ****p-val < 0.001. Statistical test: Student's *t* test. Error bars are standard error of the mean (SEM)

Altogether, the upregulation of *Hdac8* in CAF-surviving pG-2 cells, its support of the EMT program as well as its implication in patient relapse-free survival in BLBC patients strongly suggested that elevated HDAC8 levels might confer to the tumor cells chemotherapy-resistant features. To test this hypothesis, we first assessed the response of BLBC patients to cytotoxic therapies according to their *HDAC8* levels using the ROC plotter platform (Fig. 5A). In accordance with our previous results, patients showing poor responsiveness to the chemotherapy harbored higher *HDAC8* expression. In line with these data, BLBC patients with better response to cytotoxic therapies displayed significantly higher *ELF5*, *GATA3* and *RORA* expression levels (Additional file 1: Fig. S3A–C). To experimentally assess the impact of HDAC8 knockdown or inhibition in BLBC cells upon CAF treatment, we performed in vitro proliferation assays. Interestingly, loss of HDAC8 in pG-2 cells potentiated the effect of the chemotherapy on tumor cell proliferation (Fig. 5B). The increased chemotherapy efficiency was particularly visible in one resistant variant of the pG-2 cells (rG-2) [18], where siHdac8 alone had no significant effect on the cell growth (Fig. 5C). To further support the important role of HDAC8 in BLBC cell survival to chemotherapy, we simultaneously treated the murine and human cell lines with PCI-34051 and CAF. In line with the knockdown results, HDAC8 inhibition sensitized the tumor cells to the cytotoxic treatment in all cases (Fig. 5D, F). Notably, this combinatory treatment had a remarkable long-term effect on pG-2 and rG-2 cell proliferation. Finally, to translate these functional assay findings to the human situation, we investigated the effects of HDAC8 inhibition in combination with CAF chemotherapy in HCC1806 cells. Strikingly, at PCI-34051 concentrations not showing tumor cell impairment as a monotherapy, we observed a robust proliferative defect compared to the control counterparts (Fig. 5F). Collectively, our findings identified HDAC8 as a druggable factor supporting tumor cell survival to conventional chemotherapies by promoting the EMT transcriptional program in BLBC lesions.

## Discussion

EMT is a process naturally occurring in normal cells under physiologic conditions but is also frequently implicated in the gain of cancer cell aggressiveness, metastatic behavior and therapy resistance [8]. Intensive research efforts in the past decades allowed us to increase our understanding of the cascade of events, the role played by central EMT-TFs during the acquisition of mesenchymal features and their consequence for tumor cell survival to chemotherapy [12, 37]. However, to date, druggable factors regulating the EMT transcriptional program induction and their maintenance are scarce and insufficiently studied. In this context, epigenetic regulators represent attractive factors for the development of novel therapeutic approaches, as their enzymatic activity is per definition reversible and does not imply a modification of the genetic code [38]. We recently leveraged the WAP-T mammary carcinoma mouse model to get insights into transcriptional mechanisms underlying the survival of BLBC cells to a CAF chemotherapy [14]. We thereby demonstrated that the loss of PRC2 repressive activity can drive aggressive BLBC tumor cell phenotypes by promoting NFATc1 expression [18]. In the present study, we analyzed RNA-seq and ChIP-seq data from the same model and identified HDAC8 as an upregulated epigenetic factor supporting BLBC cell chemotherapy resistance by repressing the epithelial differentiation program, and with a potential as a drug target. Our results align with increasing number of studies reporting a relationship between high HDAC8 expression levels and parameters characteristic for aggressive BC [39–41]. In 2016, HDAC8 was shown to promote cell cycle progression by deacetylating the SMC3 cohesin subunit in MCF7 cells [25]. Our present investigations in BLBC malignancies identified an alternative tumor-promoting function by HDAC8 supporting the activation of EMT transcriptional program. This HDAC8-dependent mechanism is rather likely to happen in an SMC3 independent manner, as no changes of SMC3 acetylation levels were observed in tumor cells sensitized by HDAC8i treatment. Interestingly, several groups described an association between HDAC8 activity and EMT-driving signaling. Tang and colleagues reported in 2020 that HDAC8 stimulates the TGF-beta signaling by cooperating with the SMAD3/4 complex to deacetylate SIRT7 promoter region, thereby promoting BC cell migration [42]. In two consecutive studies, An et al. established a similar implication of HDAC8 controlling BC cell dissemination through the YAP signaling and via the AKT/GSK-3β/SNAI1 axis [43, 44]. Our results complement this knowledge and establish that HDAC8 can support the induction of EMT by actively repressing the expression of MET-TFs like GATA3, EFL5, RORA and GHRL2. Of note, An et al. reported a mechanism by HDAC8 enacting a stabilization of the EMT-TF SNAI1 at the protein level via phosphorylation through the AKT/GSK-3β axis [43]. *SNAI1* expression levels remained here unchanged. In contrast, we observed that loss of HDAC8 in pG-2 cells led to increased *Snai1* expression levels, both under basal culture conditions and chemotherapy treatment (data not shown). Although changes in SNAI1 protein levels were not assessed in the frame of this study, it seems reasonable to hypothesize that the loss of HDAC8 activity should dampen the increased *SNAI1* gene expression, and that parallel independent epigenetic mechanisms by HDAC8 repress the epithelial transcriptional program in a cooperative way. Importantly, the activity of HDAC8 was necessary for chemotherapy-resistant features of BLBC cells. This function is likely enacted through the repression of TFs driving the epithelial differentiation program. For example, loss of *GATA3* was associated with tumor progression and poor patient outcomes in BC and urothelial carcinoma [45, 46]. In an elegant study, Singh et al. established the ELF5 transcription factor as a tumor suppressor gene inhibiting TNBC progression and metastasis by promoting IFN-γ signaling and immunosurveillance [30]. Similarly, RORA expression was shown to possess tumor-suppressive functions and to inhibit BC tumor invasion [34]. In line, GHRL2 has emerged as an inhibitor of the EMT expression program in multiple cancer entities [35, 37, 47, 48]. Furthermore, GRHL2 expression in human mammary epithelial cells was shown to induce a metabolic switch responsible for reduction of stem cell properties and anchorage independent cell survival. We and others established in the past a strong involvement of HDACs Class I in the EMT transcriptional program activation of several cancer and fibrotic diseases [19, 49, 50]. In this context, Class I HDAC inhibitors (HDACi) showed a general good capacity to revert aggressive tumor cell phenotypes to more sensitive epithelial ones. Further investigations by Choi et al. on renal fibrosis demonstrated that Class I HDACi can revert the TGFβ1-induced repression of E-cadherin (CDH1), a major gatekeeper of the epithelial phenotype [50]. Comparing treatments with different small molecule inhibitors and siRNA-mediated knockdowns, the authors identified HDAC8 as a key factor controlling TGFβ1-mediated E-cadherin repression. Interestingly, HDAC8 enzymatic activity was not found to be necessary here. Our investigations in murine BLBC cells align with these observations, as HDAC8 knockdown in pG-2 cells rescued *Cdh1* expression upon chemotherapy. Surprisingly, inhibition of HDAC8 by PCI-34051 also led to a pronounced rescue of *Cdh1* levels, pointing at a possible context-dependent necessity of its catalytic activity to enact this repressive function.

In the past decades, growing research efforts led to the development of several pan-HDACi with very promising efficiency and FDA approval for the treatment of hematological malignancies like T-cell lymphoma and multiple myeloma [51, 52]. However, despite good results in experimental models in vitro and in vivo, the applicability of pan-HDACi compounds for the treatment of patients with solid tumors was quite deceiving, mostly because of a moderate bioavailability and relatively high toxicity at pharmacological doses, leading to severe side effects [52, 53]. Therefore, compounds targeting specific members of the HDAC family have been proposed to have a more specific anti-tumor activity, being at the same time better tolerated by patients [54]. Based on our and other studies, specific inhibitors of HDAC8 represent very attractive anti-cancer drugs. PCI-34051, used in the present work, shows a very high specificity for HDAC8 with a reported IC50 of 10 nM [55]. Its efficiency as a combination therapy with BRAF inhibitor was recently validated in an in vivo mouse model for melanoma [56]. However, the applicability of this compound as an in vivo drug has been questioned, as PCI-34051 half-life in rat brains was estimated to be 15 min only [57]. In 2015, the group of Ina Oehme successfully utilized PCI-48012, a more stable variant of PCI-34051, for the treatment of two neuroblastoma xenograft models. Strikingly, the authors observed an anti-tumor activity comparable to pan-HDACi coming along with a much lower degree of toxicity. Three years later, the same group successfully demonstrated an increased efficiency of co-treatments with AKLi and a specific HDAC8i (compound 20a) in zebrafish xenografts of neuroblastoma, thereby emphasizing the attractiveness of HDAC8i-based combined therapies.

Together, our results suggest that BLBC patients with low expression of MET-TFs may benefit from therapies combining conventional chemotherapy with HDAC8-specific inhibition.

## Conclusions

Our results establish HDAC8 as a transcriptional repressor of the MET process and as a factor targetable through epigenetic therapies with a strong potential to increase the efficiency of conventional chemotherapies.

## Methods

### Cell culture

The murine mammary carcinoma pG-2 cell line that was created in a previous study [17] as well their resistant variant (rG-2) [Mieczkowska et al. 2021, in press], was cultured in DMEM GlutaMAX™ (Invitrogen). The triple-negative breast cancer HCC1806 human cell line was purchased from the ATCC (American Type Culture Collection, USA) and was cultured in RPMI 1640 (Gibco) medium. All media were supplemented with 10% fetal bovine serum (FBS) and 1% penicillin/streptomycin (P/S), and all cell lines were maintained at 37 °C, 5% CO_2_.

### Chemotherapy treatment

CAF chemotherapy was acquired through the pharmacy of the Medical School of Göttingen at following concentrations: cyclophosphamide [20 mg/ml], doxorubicin [2 mg/ml] and 5-fluorouracil (5-FU) [50 mg/ml). In all experiments, pG-2 and rG-2 were treated with a concentration of 312.5 ng/ml cyclophosphamide, 15.6 ng/ml doxorubicin and 312.5 ng/ml 5-FU, except if differently stated in the figure legend. HCC1806 was treated with 625 ng/ml cyclophosphamide, 31.2 ng/ml doxorubicin and 625 ng/ml 5-FU, except if differently stated in the figure legend, or 2.5 nM paclitaxel or 2 µM Cisplatin. The different chemotherapies were applied for 48 h on the cells.

### siRNA transfection

Cells were reverse transfected with siRNA using Lipofectamine® RNAiMAX, according to the manufacturer’s instruction. siGENOME siRNAs (Dharmacon) were acquired at Horizon Discovery Ltd. and are listed in Additional file 1: Table S5. siRNAs: Non-targeting control #5 [D-001206–13], murine *Hdac4* [M-043626–01], murine *Hdac7:* [M-040703–01], murine *Hdac8* [M-058613–01], human *HDAC7* [D-009330–02, D-009330–04, D-009330–05, D-009330–06], human *HDAC8* [D-003500–01, D-003500–02, D-003500–03, D-003500–06]. Smart pools of 4 different siRNAs were either commercially acquired or manually mixed at an equimolar concentration of each 5 µM.

### Inhibitor treatment

Three cycles of 48-h treatment with PCI34051 (HDAC8 inhibitor, Cayman chemical, cat. no 10444) or TMP 195 (HDAC4 and HDAC7 inhibitor, Cayman chemical, cat. no 23242) were performed. 24 h after seeding, the cells were treated with one first cycle of inhibition. Chemotherapy treatment was performed together with the second cycle of inhibition.

### Cell proliferation assay

7000–10,000 cells per well were seeded in 24-well plates, depending on treatment and cell line. In case of reverse transfection with siRNA, cells were treated with chemotherapy 48 h post-transfection. The confluence was measured using a Celigo® Cell Cytometer device (Nexcelom Bioscience) and an Incucyte® Live-Cell Analysis System (Sartorius). At the last day of the experiment, cells were fixed with 100% methanol for 10 min and stained using crystal violet for 20 min. The confluency of scanned pictures was analyzed using the ImageJ software. Graphs were designed using GraphPad Prism (v8.0.1).

### RNA extraction

mRNA extraction was performed on cells cultured in 6-well plates. After removing the culture media, cells were washed twice with PBS and lysed by adding 500 µl of Qiazol®. After an incubating of 10 min at room temperature, cells were resuspended and transferred into RNase-free tubes. According to the manufacturer’s manual, 100 µl chloroform was added, vortexed for 15 s and centrifuged (12,000 g, 15 min, 4 °C). The upper aqueous phase was transferred to a new tube and 200 µl of chloroform was added, vortexed and centrifuged as before. The aqueous phase was transferred to a new tube, and mRNAs were precipitated by adding an equal amount of isopropanol (RNA grade) and incubating at − 80 °C for at least one hour. mRNAs were then pelleted (20 min/12000 g/4 °C) and washed with cold 70% ethanol (10 min,12000 g,4 °C). After removal of ethanol, the pellets were allowed to air-dry for 5 to 10 min and diluted in 30 μl of RNase-free water. RNA quantification was performed using a DS-11 + spectrophotometer (Denovix).

### cDNA synthesis

The manufacturer’s instructions in the First Strand cDNA Synthesis Kit were followed (M-MLuV, NEB). In short, 1 µg of RNA was diluted to a total volume of 10 µl with RNase-free water and mixed with 2 µl of random 9mer primers (60 µM) and 1 µl of dNTPs (10 mM). The mixture was incubated for 5 min at 65 °C, after which 2 µl of 10 × M-MuLV Buffer, 0.2 µl RNase Inhibitor (40 U/μl), 1 µl M-MuLV Reverse Transcriptase (200 U/μl) and 6.8 µl DEPC water were added and incubated at 25 °C for 5 min and then at 42 °C for 60 min. This was followed by the inactivation of the enzyme at 65 °C for 20 min. The reaction mix was diluted to a total volume of 200 µl and stored at -20 °C.

### Quantitative real-time polymerase chain reaction (qRT-PCR)

For qRT-PCR, 5 ng cDNA was utilized per reaction in 25 µl total volume (SYBR-green). Samples were run with the following steps: denaturation (95 °C for 2 min), 40 cycles (for gene expression) or 46 cycles (for ChIP-RT-PCR) amplification (95 °C for 15 s followed by 60 °C for 30 s). A melting curve analysis was subsequently generated (60 °C to 95 °C, 0.5 °C per sec). Samples were quantified by using the method of standard curve. The samples were normalized to the *Rplp0* or *RPLP0* housekeeping genes. Graphs were designed using GraphPad Prism (v8.0.1).

### Western blotting

Proteins were extracted from six-well plates with 500 µl ice-cold RIPA buffer (10 mM Tris–HCl pH 8, 1 mM EDTA, 1% v/v Triton X-100, 0.1% sodium deoxycholate, 0.1% SDS, 140 mM NaCl) supplemented with protease inhibitors (1 mM Pefabloc, 1 ng/µl aprotinin/leupeptin, 10 mM BGP, 1 mM NEM). Protein samples were then sonicated three times for five cycles (30 s ON/30 s OFF) in a Bioruptor Pico (Diagenode, Belgium). Lysates were then mixed with Lämmli buffer (6 × , 375 mM Tris–HCl, 10% SDS, 30% glycerol, 0.02% bromophenol blue, 9.3% DTT) and cooked for 5 min at 95 °C. Same amounts of protein per sample were then separated using 10–12% SDS polyacrylamide gel electrophoresis and transferred onto nitrocellulose membranes (Immobilon, Millipore, USA). Membranes were blocked with 5% skimmed milk in TBS-T and incubated overnight at 4 °C with specific primary antibodies diluted in the same blocking solution. Next, membranes were washed with TBS-T, incubated 1 h with secondary antibodies (Dianova GmbH, Germany) diluted in blocking buffer, washed again with TBS-T and finally developed using HRP substrate (Cyanogen WESTAR) in a ChemoStar imaging system (INTAS science imaging, Germany). Antibody list in Additional file 1: Table S6.

### RNA sequencing analysis

RNA-seq raw data (Fastq files) were processed in the GWDG Galaxy environment (https://galaxy.gwdg.de) provided by the “Gesellschaft für wissenschaftliche Datenverarbeitung mbH Göttingen” (GWDG). After quality check using FastQC (v0.72) [58], sequencing data were trimmed (FASTQ Trimmer tool, v0.0.1), aligned to the murine reference genome (mm9) with the TopHat tool (v2.1.1) [59]. Next, reads were assigned to their respective genomic features using htseq-count (v0.1.9) [60]. Finally, a differential gene expression analysis was performed using DESeq2 (v2.11.40.5) [61]. Analyses of gene signature enrichment were performed using the Gene Set Enrichment Analysis (GSEA, v4.1.0) tool (http://www.broadinstitute.org/gsea/downloads.jsp) and online pathway enrichment analysis tool Enrichr (http://amp.pharm.mssm.edu/Enrichr/) as well as gProfiler (https://biit.cs.ut.ee/gprofiler/gost). Heatmap representations of gene expression were generated with the online Morpheus tool (https://software.broadinstitute.org/morpheus/). RNA-seq raw data are deposited at ArrayExpress (https://www.ebi.ac.uk/arrayexpress/) under the accession number E-MTAB-9547.

### Chromatin immunoprecipitation (ChIP)

Chromatin immunoprecipitation for H3K27me3 and H3K27ac was performed 48 h after chemotherapy treatment, as described previously [62]. Briefly, pG-2 cells were cultured in 15 cm plates. Protein–DNA complexes were crosslinked with 1% formaldehyde (Sigma), and nuclear fraction was extracted and sonicated with a Bioruptor pico (Diagenode). After controlling the size of the DNA fragments and a pre-cleaning step, the same amounts of samples were incubated with 1 µg anti-H3K27me3 or anti-H3K27ac antibody overnight at 4 °C and immunoprecipitated with protein A-sepharose. Finally, DNA–protein complexes were reverse-crosslinked, DNA fragments were purified by phenol–chloroform extraction, and concentration was determined using Qubit fluorimeter (Invitrogen).

### Chromatin immunoprecipitation-sequencing analysis

ChIP-seq data were processed and analyzed in the Galaxy environment (https://galaxy.gwdg.de/). After a quality check (FastQC, v0.72), reads were aligned to the mouse reference genome (mm9) using Bowtie2 [58, 63]. H3K27ac peaks were identified with the MACS2 tool (v 2.1.1.20160309.0), and Differential Binding Analysis as well volcano plots were performed with Diffbind (Bioconductor, v3.6.3) [64]. The deep tools suite was used for the generation of normalized coverage files (bamcoverage, v3.2.0.0.0) [65]. To visualize occupied regions, region scoring matrix was computed (computeMatrix, v3.2.0.0.0) and profiles plots or heatmaps were generated [plotProfile (v3.2.0.0.0) and plotHeatmap (v3.2.0.0.1)] [65]. Histone modification occupancy at specific genomic regions was visualized with the integrative genome Viewer (IGV, v2.8.0, http://software.broadinstitute.org/software/igv/). Pathway enrichment analysis of differentially regulated genomic regions was performed using the Genomic Regions of Annotations Tool (GREAT, http://great.stanford.edu/public/html/). ChIP-seq raw data are deposited at ArrayExpress (https://www.ebi.ac.uk/arrayexpress/) under the accession number E-MTAB-9584.

List of EMT and MET genes in Fig. 3D: EMT genes were extracted from MSigDB C2: HOLLERN_EMT_BREAST_TUMOR_UP, VERHAAK_GLIOBLASTOMA_MESENCHYMAL and JECHLINGER_EPITHELIAL_TO_MESENCHYMAL_TRANSITION_UP; MET genes were extracted from MSigDB C2: HOLLERN_EMT_BREAST_TUMOR_DN). The murine transcription factor list was retrieved from the Animal TFDB3.0 TF database (source: http://bioinfo.life.hust.edu.cn/AnimalTFDB/#!/).

### Publically available data

Overall survival data of BLBC patients from The Cancer Genome Atlas (TCGA) program (https://portal.gdc.cancer.gov/) were retrieved from the xenabrowser web-based tool (http://xena.ucsc.edu/) using the following patient selection criteria: PAM50 subtype (Nature, 2012): basal-like, ER, PR, HER2 status (Nature, 2012): negative. The *Cutoff Finder* online tool (https://molpathoheidelberg.shinyapps.io/CutoffFinder_v1/) was used to determine the best cutoffs and the corrected *p*-values [66].

Relapse-free survival data of BLBC patients the online KM plotter tool (https://kmplot.com) were analyzed in the dedicated web-based platform [67]. Used datasets: 223345_at (*HDAC8*), 220624_s_at (*ELF5*), 209602_s_at (*GATA3*) and 240951_at (*RORA)*. Used patient selection criteria: PAM50: basal-like, endocrine therapy excluded, only neoadjuvant therapy. Overall survival data of BLBC patients were acquired from the same online tool. Used datasets: 228813_at (*HDAC4*), 217937_s_at (*HDAC7*), 223345_at (*HDAC8*). Used patient selection criteria: PAM50: basal-like, endocrine therapy excluded, any chemotherapy.

Finally, gene expression data of *HDAC8* and the MET-TFs for Fig. 4D, E were retrieved from the online database tool “R2 Genomic Analysis and Visualization Platform” (R2.amc.nl). Graphs were designed using GraphPad Prism (v8.0.1).

Receiver operating characteristic (ROC) patient data were retrieved from the ROC plotter (source: https://rocplot.org/site/treatment) using the relapse-free survival (RFS; at 5 years) and pathological complete response (pCR) data. RFS: 223345_at (*HDAC8*), 209604_s_at (*GATA3*). pCR: 220625_s_at (*ELF5*), 1562682_at* (*RORA*). Patient selection criteria: TNBC patients, receiving any chemotherapy.

Gene expression microarray data of siControl- and siSMC3-treated MCF7 cells were retrieved from the Gene Expression Omnibus database (https://www.ncbi.nlm.nih.gov/geo/) under the accession number GSE38252 [68], re-analyzed with the GEO2R tool (https://www.ncbi.nlm.nih.gov/geo/geo2r/) and visualized in a volcano plot using R (v 4.1.1).

## Supplementary Information

**Additional file 1: Figure S1**. HDAC8 supports the induction of EMT by suppressing the MET pathway in TNBC cells surviving Chemo. **Table S1**. Concentration of CAF dilutions. **Table S2.** List of ChIP-qPCR primers. **Table S3.** List of RT-qPCR mouse primers. **Table S4.** List of RT-qPCR human primers, excel file with the list of epigenetic regulators. **Table S5.** List of siRNAs.

## Data Availability

Not applicable.

## Abbreviations

BLBC: Basal-like breast cancer
TNBC: Triple-negative breast cancer
EMT: Epithelial–mesenchymal transition
MET: Mesenchymal–epithelial transition
HDAC8: Histone deacetylation 8
TF: Transcription factor
H3K27ac: Acetylation of lysine 27 on histone H3
CAF: Cyclophosphamide, adriamycin, 5-fluorouracil
