# Supplementary material for: HDAC8 suppresses the epithelial phenotype and promotes EMT in chemotherapy-treated basal-like breast cancer
Source: Clin Epigenetics. 2022 Jan 11;14:7. doi: 10.1186/s13148-022-01228-4 (PMC8753869; doi:10.1186/s13148-022-01228-4)
Supplement: Supplementary file 1 — Additional file 1: Figure S1. HDAC8 supports the induction of EMT by suppressing the MET pathway in TNBC cells surviving Chemo. Table S1. Concentration of CAF dilutions. Table S2. List of ChIP-qPCR primers. Table S3. List of RT-qPCR mouse primers. Table S4. List of RT-qPCR human primers, excel file with the list of epigenetic regulators. Table S5. List of siRNAs. [file 13148_2022_1228_MOESM1_ESM.docx]

**Additional Informations: Pantelaiou-Prokaki et al.**

**Title**

**HDAC8 suppresses the epithelial phenotype and promotes EMT in chemotherapy-treated basal-like breast cancer**

Garyfallia Pantelaiou-Prokaki^1,2#^, Iga Mieczkowska^3#^, Geske E. Schmidt^4^, Sonja Fritzsche^1^, Evangelos Prokakis^1^, Julia Gallwas^1^, Florian Wegwitz^1,^*

^1^Department of Gynecology and Obstetrics, University Medical Center Göttingen, Göttingen, Germany

^2^Translational Molecular Imaging, Max Planck Institute for Experimental Medicine, Göttingen, Germany

^3^Department of General, Visceral and Pediatric Surgery, University Medical Center Göttingen, Göttingen, Germany

^4^Department of Gastroenterology, GI-Oncology and Endocrinology, University Medical Center Göttingen, Göttingen, Germany

# equal author contribution

* Correspondence: [fwegwit@gwdg.de](mailto:fwegwit@gwdg.de)

**
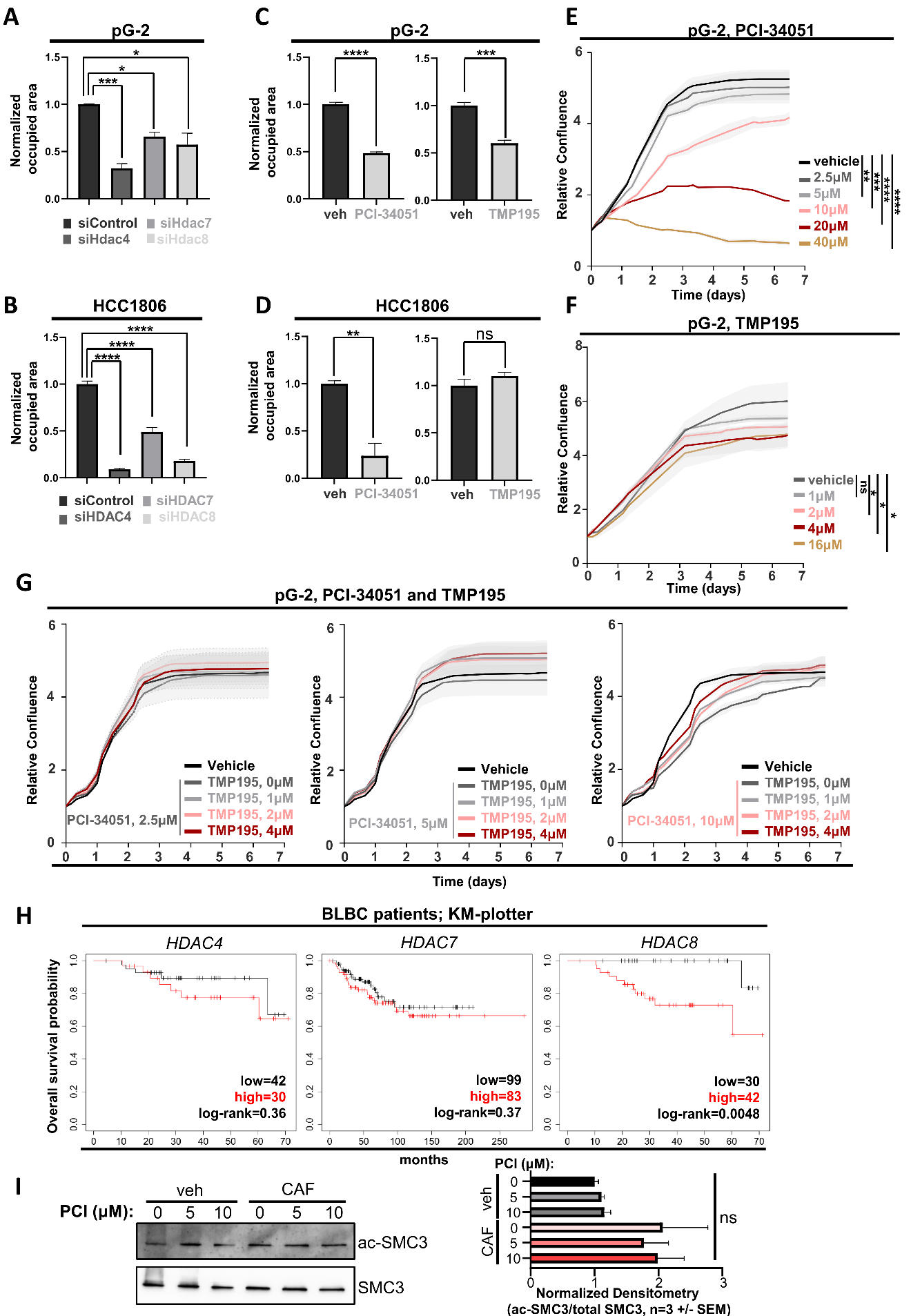
**

**Figure S1: HDAC8 inhibition is detrimental to the cell viability of BLBC cells.
A-B)** Relative area quantification of control-, HDAC4-, HDAC7- and HDAC8-silenced pG-2 **(A)** and HCC1806 **(**n=4) **(B)** cells. The data are calibrated on the respective control conditions **C-D)** Relative area quantification of veh (vehicle), PCI-34051 and TMP195-treated pG-2 **(C)** and HCC1806 **(D)** cells. The data are calibrated on the respective vehicle conditions **E-G)** Proliferation assay of pG-2 cells with increasing doses of PCI-34051 **(E)**, TMP195 **(F)** and PCI-34051 and TMP195 in combination **(G)** assessed with an Incucyte®. Data normalized to the time point 0 **H)** Kaplan-Meier plots showing the overall survival probability of *HDAC4*-, *HDAC7*- and *HDAC8*-expressing BLBC patients (KM-plotter). **I)** Western blot analysis of ac-SMC3 and SMC3 protein levels upon treatment with PCI-34051 (PCI, 5μΜ or 10μM) on vehicle and CAF-treated pG-2 cells. Statistical test: **A-B,I:** One-way Anova, **C-D:** student t-test. **E-F:** One-way Anova on the area under the curve (AUC). ns= not significant * p-val<0.05, ** p-val<0.01, *** p-val<0.005, **** p-val<0.0001. Error bars: standard error of the mean (SEM). All experiments were performed at least in biological triplicates.

**
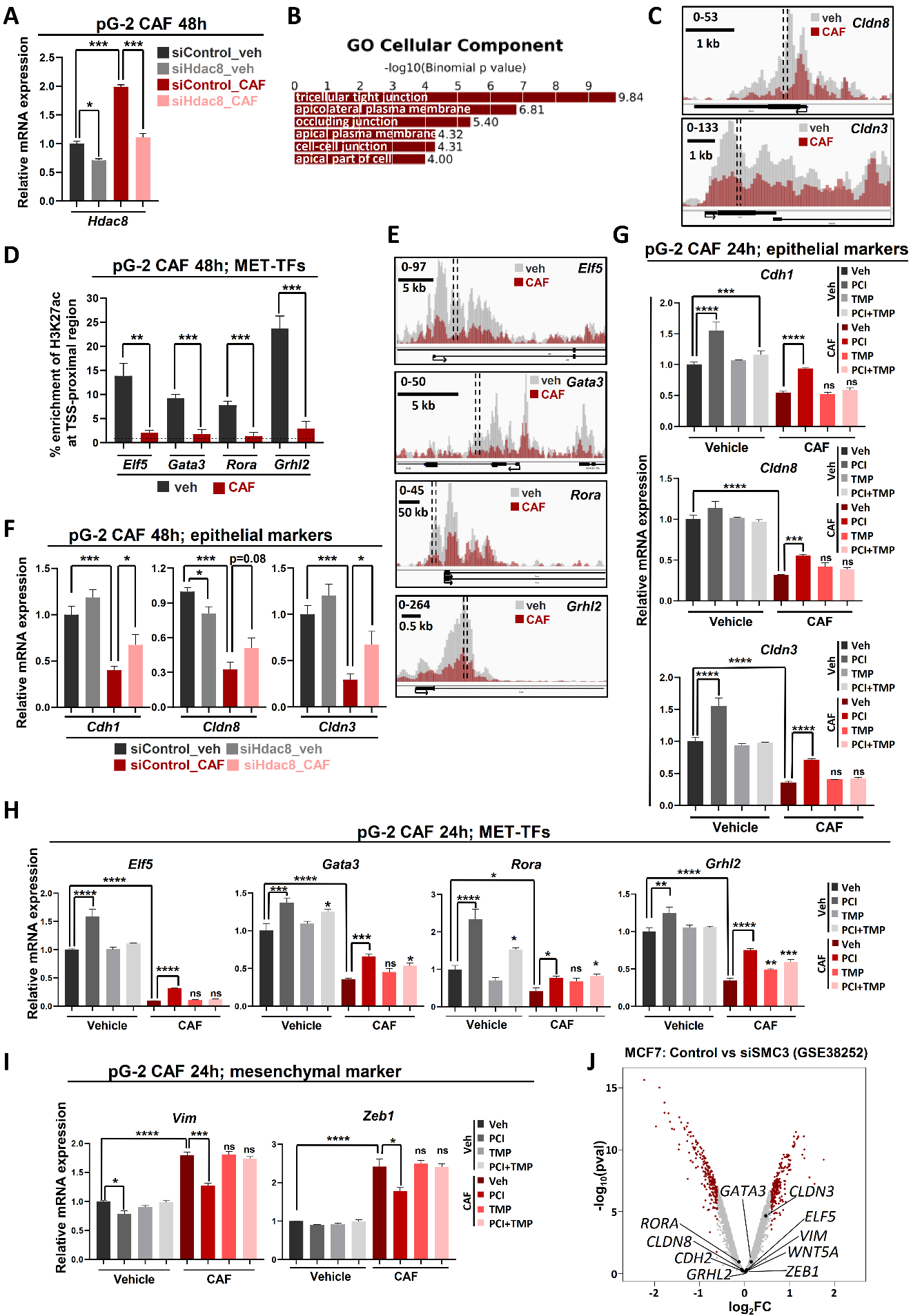
**

**Figure S2: HDAC8 supports the induction of EMT by suppressing the MET pathway in TNBC cells surviving chemo. A)** *Hdac8* mRNA expression in CAF-treated (48h) pG-2 cells upon *Hdac8* silencing. **B)** Pathway enrichment analysis of TSS-associated genomic region losing H3K27ac showing MET- and cell differentiation-associated signatures using the online tool Genomic Regions Enrichment of Annotations Tool (GREAT). **C)** H3K27ac occupancy changes in the TSS-genomic regions of selected MET-markers *Cldn8* and *Cldn3* upon 48h CAF treatment of pG-2 cells. The dashed lines display the binding sites for the ChIP-qPCR primer pairs used in the subsequent experiments. **D)** ChIP-qPCR at TSS-regions of the MET-TFs *Elf5*, *Gata3*, *Rora*, *Grhl2* upon 48h CAF treatment of pG-2 cells. **E)** H3K27ac occupancy changes in the TSS-proximal regions of selected MET-TFs *Elf5*, *Gata3*, *Rora*, *Grhl2* upon 48h CAF treatment of pG-2 cells. The dashed lines display the binding sites for the ChIP-qPCR primer pairs. **F)** qRT-PCR of epithelial markers *Cdh1, Cldn8, Cldn3* in CAF-treated pG-2 cells upon *Hdac8* silencing. **G-I)** qRT-PCR of epithelial markers *Cdh1*, *Cldn8*, *Cldn3* **(G),** of MET-TFs *Elf5*, *Gata3*, *Rora, Grhl2* **(H)** and of the mesenchymal markers *Vim, Zeb1* **(I)** on vehicle and CAF-treated pG-2 cells upon inhibition with PCI-34051 (PCI, 10μΜ), or inhibition with TMP195 (TMP, 8μM), or inhibition with the combination of PCI-34051 and TMP195 (PCI+TMP, 10μΜ+8μM). **J)** Volcano plot of publicly available microarray data (GSE38252) showing that EMT and MET genes that are not regulated upon SMC3 silencing in MC7 BC cells. All experiments were performed in biological triplicates. * p-val<0.05, ** p-val<0.01, *** p-val<0.005. Statistical tests: Student t-test (**D**), One-way Anova (**A, F-I**). Error bars are standard error of the mean (SEM).

**
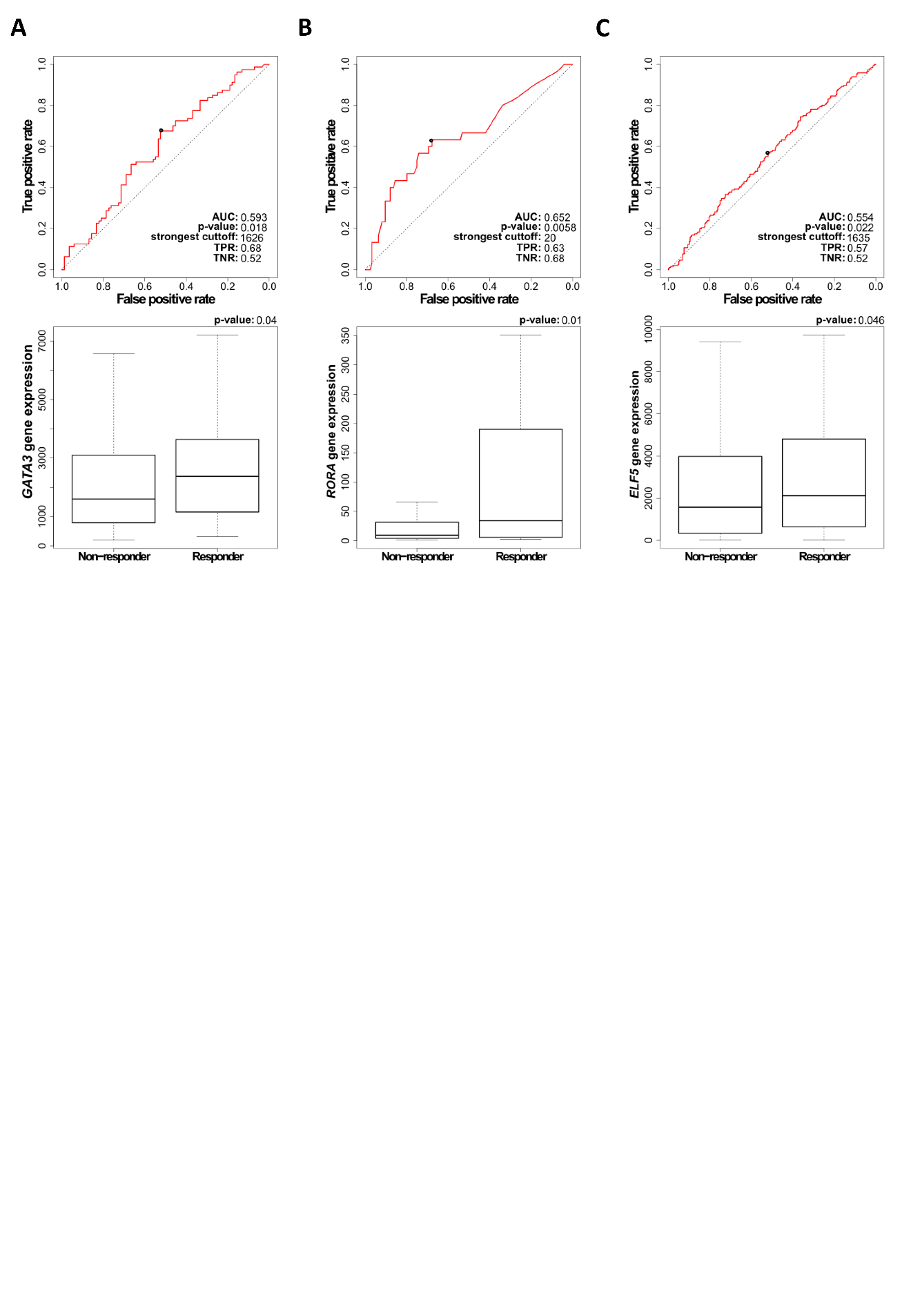
**

**Figure S3: High expression of gatekeeper transcription factors of the epithelial phenotype support the responsiveness to conventional chemotherapy in TNBC patients. A-C)** ROC analysis from publically available TNBC data demonstrate an improved response to chemotherapy in *GATA3*^high^ **(A)**, *RORA*^high^ **(B)** and *ELF5*^high^ TNBC patients **(C)**. Box plots: Mann Whitney test.

**Table S1: Concentration of CAF dilutions.**

|  | **Concentration (µg/ml)** | **Dilutions (µg/ml)** | | |
| --- | --- | --- | --- | --- |
| **CAF** | **1** | **1/16(high)** | **1/32(IC50)** | **1/64(low)** |
| Cyclophosphamide | 10 | 0,625 | 0.3125‬ | 0.15625‬ |
| Doxorubicin | 0.5 | 0,03125‬ | 0.015625 | 0.0078125‬ |
| 5-Fluorouracil | 10 | 0,625 | 0.3125‬ | 0.15625‬ |

**Table S2: List of ChIP-qPCR primers.**

| *Elf5* | forward | ACAAGAAGGCCCGGTAATTT |
| --- | --- | --- |
| *Elf5* | reverse | CGAGAGAATGAAGGCAGGAG |
| *Cldn3* | forward | CCAACTGCGTACAAGACGAG |
| *Cldn3* | reverse | ATCCCTGATGATGGTGTTGG |
| *Cldn8* | forward | GTGCACTTCATTCCGAGGAT |
| *Cldn8* | reverse | AGGTCTACGACTCCCTGCTG |
| *Grhl2* | forward | GAATGGGGGACTGATTTTGA |
| *Grhl2* | reverse | GACGCGTATGAGCCAGAGAC |
| *Elf3* | forward | TTGAGCCTAGAAGGGTCAGG |
| *Elf3* | reverse | GGAGAGAGCCCCAGAAGAAT |
| *Gata3* | forward | TAGCCGGGAGTCTAAGCAAA |
| *Gata3* | reverse | GCCCAAATACGTGCATTTCT |
| *Rora* | forward | CGACCGATCCTTTCTGTAGC |
| *Rora* | reverse | ACACTCTCCACCCTCCAGAC |

**Table S3: List of RT-qPCR mouse primers.**

| *Hdac7* | forward | TCTCTTCCTGGCAGGCTTAC |
| --- | --- | --- |
| *Hdac7* | reverse | AGTTGCCGAAGTTCTTGCTC |
| *Hdac8* | forward | AGGTGATGAGGACCATCCAG |
| *Hdac8* | reverse | ACCCTCCAGACCAGTTGATG |
| *Hdac4* | forward | CTGTGGAGCTGCTGAATCCT |
| *Hdac4* | reverse | TTCCAAGGGCAGTGAGAACT |
| *Vim* | forward | CGGCTGCGAGAAATTGC |
| *Vim* | reverse | CCACTTTCCGTTCAAGGTCAAG |
| *Zeb1* | forward | CACCAGAAGCCAGCAGTCAT |
| *Zeb1* | reverse | CGTTCTTCTCATGGCGGTACT |
| *Cdh2* | forward | AGAGCACATGCAGTGGACATC |
| *Cdh2* | reverse | GGCAGTGACCGTCATCACATA |
| *Snai1* | forward | CTGGTGAGAAGCCATTCTCCT |
| *Snai1* | reverse | CCTGGCACTGGTATCTCTTCA |
| *Cdh1* | forward | AGCCATTGCCAAGTACATCCTC |
| *Cdh1* | reverse | GGCCTGTTGTCATTCTGATCTG |
| *Elf5* | forward | CTACCCTGCCTTTGAGCATC |
| *Elf5* | reverse | TACTGGTCGCAGCAGAATTG |
| *Cldn8* | forward | TCCCAAGGCGTACAGATTTC |
| *Cldn8* | reverse | CAGTGCAGCCATTTGAAGAG |
| *Grhl2* | forward | AAAAGGGGAGCGAGTTCATT |
| *Grhl2* | reverse | GTAGGCTCTTCGGGTGTTGA |
| *Cldn3* | forward | GAGATGGGAGCTGGGTTGTA |
| *Cldn3* | reverse | GGATCTTGGTGGGTGCATAC |
| *Rora* | forward | CTCGCTGAAGTCAAGGAAGG |
| *Rora* | reverse | CCGTGACTACCATGGTTCCT |
| *Gata3* | forward | CCCTCCGGCTTCATCCTCT |
| *Gata3* | reverse | CTGCACCTGATACTTGAGGC |
| *Wnt5a* | forward | GGAACGAATCCACGCTAAGG |
| *Wnt5a* | reverse | GGAGCCAGACACTCCATGAC |

**Table S4: List of RT-qPCR human primers.**

| *VIM* | forward | TACAGGAAGCTGCTGGAAGG |
| --- | --- | --- |
| *VIM* | reverse | ACCAGAGGGAGTGAATCCAG |
| *CDH2* | forward | AGGTTTGCCAGTGTGACTCC |
| *CDH2* | reverse | CCACAAACATCAGCACAAGG |
| *SNAI1* | forward | GCGAGCTGCAGGACTCTAAT |
| *SNAI1* | reverse | GGACAGAGTCCCAGATGAGC |

**Table S5: List of siRNA.**

| **Gene** | **Sequence (5’-3’)** | **Cat.no** | **Company** |
| --- | --- | --- | --- |
| NT5  (Non-targeting control #5) | UGGUUUACAUGUCGACUAA | D-001206-13 | Dharmacon |
| *Hdac4* | GCUCAAGGCUUAAGCAGAA  CCAAGAAACUUACCCGUAU  GCAGAGGAUCCACCAGUUA  GUGGAUAGCGACACCAUAU | M-043626-01 | Dharmacon |
| *Hdac7* | CCGAAAGGCUUCCCUAGAG  UGACGCAGCAGUUGAUGAA  GCUACAGCAACACGGCAAA  GAGUGGGACCUAUGGCGAA | M-040703-01 | Dharmacon |
| *Hdac8* | CAUCGAAGGUUAUGACUGU  GACGGGAAGUGUAAAGUAG  CUACGUGGAUUUGGAUCUA  CUGAUUAUGUGCUGGAAAU | M-058613-01 | Dharmacon |
| *HDAC7* | GGAAGAACCUAUGAAUCUC  GAAGCUAGCGGAGGUGAUU  GACAAGAGCAAGCGAAGUG  AGAAUCCACUGCUCCGAAA | D-009330-02  D-009330-04  D-009330-05  D-009330-06 | Dharmacon |
| *HDAC8* | GCUGGGAGCUGACACAAUA  GGAAUUGGCAAGUGUCUUA  GCAAGUGUCUUAAGUACAU  GACGGAAAUUUGAGCGUAU | D-003500-01  D-003500-02  D-003500-03  D-003500-06 | Dharmacon |

**Table S6: List of Antibody.**

| **Antibody** | **Western Blot** | **Cat.no** | **Company** |
| --- | --- | --- | --- |
| SMC3 | 1:500 | #5696S | Cell Signaling Technology |
| acSMC3 | 1:500 | #PD040 | MBL international corporation |
